# The Effect of Multi‐Strain Probiotics, Zinc and Copper on the Performance of Broiler Chickens Challenged With *Clostridium perfringens*

**DOI:** 10.1002/vms3.71176

**Published:** 2026-09-19

**Authors:** Amin Kazemi, Hassan Kermanshahi, Reza Majidzahe Heravi, Gholamreza Salehi Jouzani, Ali Javadmanesh

**Affiliations:** ^1^ Department of Animal Science, Faculty of Agriculture Ferdowsi University of Mashhad Mashhad Iran; ^2^ Microbial Biotechnology Department Agricultural Biotechnology Research Institute of Iran (ABRII) Karaj Iran

**Keywords:** broiler, *Clostridium perfringens*, copper, probiotics, zinc

## Abstract

**Background:**

*Clostridium perfringens* infection is a major cause of necrotic enteritis in broiler chickens, leading to impaired growth performance and intestinal health. Nutritional strategies, such as probiotics and chelated trace minerals, may help mitigate these adverse effects.

**Objectives:**

This study investigated the effects of multi‐strain probiotics and chelated forms of zinc and copper on the performance, gut health and physiological responses of broiler chickens challenged with *C. perfringens*.

**Methods:**

A completely randomized design with six treatments involving 390 male broiler chickens was used. Treatments included (1) control, (2) control + challenge with *C. perfringens*, (3) challenge + selected probiotic strains, (4) challenge + chelated zinc, (5) challenge + chelated copper and (6) challenge + a combination of probiotics, zinc and copper. Growth performance, carcass characteristics, gut histomorphology, ileal pH, microbial populations, blood biochemical parameters and volatile fatty acids (VFAs) were evaluated.

**Results:**

*C. perfringens* infection significantly (*p* < 0.05) impaired growth performance and gut health, as evidenced by reduced body weight gain, poorer feed conversion ratio and alterations in intestinal morphology. Probiotic supplementation significantly improved growth performance, indicated by higher body weight at Days 1–10 (*p* = 0.048) and 1–21 (*p* = 0.020). It also enhanced intestinal morphology, including increased villus height and villus width (*p* < 0.05), and modulated gut microbial balance in the ileum by increasing *Bacillus* counts (*p* = 0.004) and decreasing *C. perfringens* populations (*p* < 0.001). Furthermore, dietary supplementation with probiotics, chelated zinc and chelated copper, individually or in combination, mitigated the adverse effects of infection by improving growth performance, supporting gut integrity and enhancing antioxidant status, as indicated by reduced malondialdehyde (MDA) levels.

**Conclusions:**

Supplementation with probiotics and chelated zinc during necrotic enteritis challenge can serve as an effective nutritional strategy to alleviate the detrimental effects of *C. perfringens* infection and improve intestinal health in broiler chickens.

## Graphical abstract

Our study showed that challenging broiler chickens with *Clostridium perfringens* reduced performance and increased mortality. Conversely, supplementing the diet with additives, such as chelated zinc, chelated copper and probiotics, helped mitigate the negative effects of *C. perfringens*. In general, it can be concluded that the use of these additives reduces the potential costs associated with necrotic enteritis in poultry and helps ensure bird health

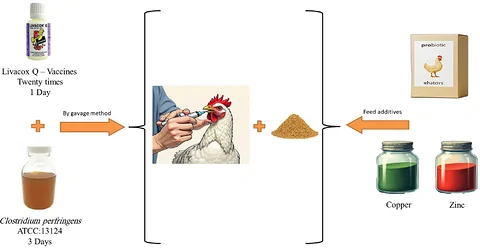

## 1 Introduction

The ban on using antibiotics as growth promoters in poultry feed has led to an increase in bacterial diseases and related mortality rates (Rafiq et al. 2022). This situation has driven researchers to seek alternative methods to replace antibiotics, ensuring the safety of both livestock and humans (Shehata et al. 2025; Luna et al. 2025). As the use of antibiotics decreases, infectious intestinal diseases have risen, placing significant economic pressure on the global poultry industry (Abd El‐Hack et al. 2022).

Among these diseases, necrotic enteritis has emerged as a particularly challenging issue. One of the most important pathogens associated with intestinal diseases in poultry is *Clostridium perfringens*, a Gram‐positive, anaerobic, spore‐forming bacterium widespread in nature and responsible for various diseases in both humans and animals (Daneshmand et al. 2020; Fu et al. 2022). Under normal conditions, *C. perfringens* is present in low numbers in the gut microbiota and does not impair bird performance; however, several predisposing factors, such as coccidiosis, diets rich in non‐starch polysaccharides like wheat, and animal protein sources, can promote its proliferation and toxin production, leading to necrotic enteritis (Fancher et al. 2020; Pietruk et al. 2025; Bedford and Apajalahti 2022). This disease adversely affects poultry health and performance, causing significant reductions in feed intake and weight gain (Villagrán‐de la Mora et al. 2020; Remus et al. 2014). *C. perfringens* is classified into five toxinotypes (A–E), with Types A and occasionally C primarily responsible for necrotic enteritis in birds (Gohari and Prescott 2022; Mehdizadeh Gohari et al. 2021). Stress in birds creates a digestive environment conducive to the proliferation of pathogenic bacteria (Obianwuna et al. 2023; Wickramasuriya et al. 2022).

Given these challenges, researchers have focused on nutritional strategies as viable alternatives to antibiotic growth promoters. Various feed additives, including probiotics, prebiotics, enzymes, immune stimulants, plant oils, chelated acids and chelated minerals, have been applied in poultry nutrition to improve gut health and performance, as demonstrated in recent studies (Alqhtani et al. 2024: Alkhulaifi et al. 2022). Probiotics are live microorganisms administered in appropriate amounts that improve the health status of the host (Abd El‐Hack et al. 2022). These microorganisms help regulate gut microbiota, improving host health and nutrient digestion, while producing antimicrobial substances that suppress pathogenic bacteria through mechanisms such as competitive exclusion (Fathima et al. 2022b, 2022a; Aliyi 2023). The major microorganisms used in probiotic production include bacteria from the *Bacillus, Lactobacillus, Bifidobacterium, Streptococcus* and *Pediococcus* families, as well as *Saccharomyces* yeast and certain strains of *Aspergillus oryzae* (Arsène et al. 2021; Lambo et al. 2021). In addition, probiotic microorganisms may produce digestive enzymes such as amylase, trypsin and lipase, thereby enhancing gastrointestinal function (Assan et al. 2022).

Mineral supplementation has also been explored for its role in improving gut health and performance under pathogenic challenge. Zinc plays a critical role in maintaining intestinal integrity and immune function during pathogenic challenges. Zinc, an essential mineral for animals, can reduce intestinal permeability and decrease inflammation associated with necrotic enteritis (Bortoluzzi, Vieira, et al. 2019). In poultry, serum and plasma zinc concentrations decrease during infections such as those caused by *Salmonella* and *C. perfringens*. This suggests that higher dietary zinc may be necessary when intestinal absorption is compromised due to infections like coccidiosis and necrotic enteritis (Sun et al. 2020). Supplementing zinc in the diet of broiler chickens affected by *C. perfringens* has been reported to improve performance, enhance the restoration of intestinal cell membrane integrity and moderate cytokine expression (Bortoluzzi, Lumpkins, et al. 2019).

Similarly, copper has received attention as a performance‐enhancing additive in poultry nutrition. Copper supplementation has garnered significant attention from researchers due to its potential to improve feed conversion ratios (Bortoluzzi et al. 2020). One of the earliest reports on copper supplementation in poultry feed demonstrated its positive effects on growth stimulation (Mehring et al. 1960). These authors found that feed efficiency improves when poultry feed is supplemented with copper, indicating effects similar to antibiotics. Additionally, Pang et al. (2009) reported positive effects of using copper in broiler diets, showing that supplementing copper sulphate at 250 mg/kg of feed reduces the population of *Escherichia coli* in the gut of broiler chickens.

Although numerous studies have independently demonstrated the beneficial effects of probiotics, zinc or copper on gut health and performance in broilers, most investigations have evaluated these additives in isolation. Limited information is available regarding the combined application of multi‐strain probiotics with chelated zinc and copper under conditions of *C. perfringens* challenge. Given the potential complementary mechanisms of these additives in modulating gut microbiota, intestinal integrity and immune responses, evaluating their combined effects may provide a more effective nutritional strategy for controlling necrotic enteritis. Therefore, the present study was conducted to investigate the effects of multi‐strain probiotics, zinc and copper, alone and in combination, on the performance of broiler chickens challenged with *C. perfringens*.

## 2 Materials and Methods

The experimental procedures and animal care protocols used in this study were reviewed and approved by the Ethics and Animal Welfare Commission of Ferdowsi University.

### 2.1 Broiler Chicks

This stage employs a completely randomized design with six treatments, using a total of 390 male broiler chickens from a commercial strain (ROSS 308). Each treatment included 5 replicates with 13 broiler chickens per replicate. The study was conducted until the chickens reached 21 days of age (Table 1). The treatments were as follows:

1. Control: Broiler chickens fed a diet without any additive.
2. Control + *C. perfringens* challenge: Control group challenged with *C. perfringens*.
3. Control + *C. perfringens* challenge + selected strain mix: Control group challenged with *C. perfringens* and supplemented with a mix of selected probiotic strains (500 g/t of feed with a concentration of 10^9^ CFU/g).
4. Control + *C. perfringens* challenge + chelated zinc: Control group challenged with *C. perfringens* and supplemented with chelated zinc (120 mg/kg).
5. Control + *C. perfringens* challenge + chelated copper: Control group challenged with *C. perfringens* and supplemented with chelated copper (150 mg/kg).
6. Control + *C. perfringens* challenge + chelated copper + chelated zinc + probiotic strains: Control group challenged with *C. perfringens* and supplemented with chelated copper, chelated zinc and a mix of selected probiotic strains.

**TABLE 1:** Diagram of classical experimental design.

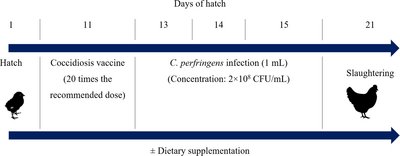

*Note*: Chickens were fed with basal diet with probiotics, zinc and copper co‐infected with *coccidiosis* on Day 11 of the experiment and *Clostridium perfringens* on Days 13, 14 and 15 post‐hatch, respectively.

Throughout the experiment, broiler chickens had ad libitum access to both water and feed. The dietary formulation for each experimental group was based on the guidelines of the National Research Council (NRC 1994) (Table 2).

**TABLE 2:** Ingredient, composition and nutrient content of experimental diets.

Item	Starter (1–10 days)	Grower (11–21 days)
**Ingredients, %**		
Corn	53.43	57.3
Soybean meal, 440 g of CP/kg	40.00	36.00
Soy oil	2.00	2.56
Dicalcium phosphate	1.95	1.72
Caco3	1.07	0.90
Nacl	0.17	0.18
NaHco3	0.29	0.27
DL Met	0.31	0.26
L Lys	0.18	0.14
L Thr	0.10	0.07
Vitamin and mineral mixture^a^	0.50	0.50
**Calculated chemical composition** ^b^		
Metabolisable energy, kcal/kg	2868	2958
Crude protein, %	22.03	20.48
Lysine, %	1.21	1.10
Methionine + Cysteine, %	0.90	0.83
Threonine, %	0.82	0.74
Calcium, %	0.96	0.87
Phosphorus (available), %	0.48	0.43

^a^Supplied per kilogram of feed—vitamin A; 12,500 IU, vitamin D3; 1250 IU, vitamin E; 18 IU, vitamin K3; 3.7 mg, thiamine; 1.8 mg, riboflavin; 6.6 mg, calcium pantothenate; 10 mg, niacin; 37.5 mg pyridoxine; 32.5 mg, vitamin B12; 2.5 mg, Mn; 50 mg, Zn; 37.5 mg, Fe; 25 mg, Cu; 7.5 mg.

^b^ According to National Research Council (1994).

### 2.2 Induction of Challenge

On the basis of the method described by Wu et al. (2014), with slight modifications, after placing the chickens in the experimental hall and randomly assigning them to treatments, a coccidiosis vaccine was administered via oral gavage into the crop to all groups except the negative control group on Day 11 of the experiment. The negative control groups received 0.2 mL of saline solution via oral gavage into the crop. A standard dose (0.01 mL) of the coccidiosis vaccine LIVACOX Q (a product from the Czech Republic) contains 300–500 live sporulated oocysts of each attenuated line of *Eimeria acervulina*, *Eimeria maxima* and *Eimeria tenella*, and 100 live sporulated oocysts of the attenuated line of *Eimeria necatrix*, suspended in a 1% w/v aqueous solution of Chloramine B. To prepare the 20× recommended dose, each 1 mL of vaccine solution, originally sufficient for 100 birds (0.01 mL per bird), was diluted with 9 mL of distilled water to yield a 10 mL solution for 100 birds. Subsequently, each bird received 2 mL of this diluted solution via oral gavage, corresponding to 20 times the standard dose.

On Days 13, 14 and 15, all chickens except those in the negative control group received 1 mL of a *C. perfringens* liquid culture (concentration: 2 × 10^8^ CFU/mL) via oral gavage into the crop. The negative control group received 1 mL of saline solution via the same route on the same days. To prevent cross‐contamination, pens housing the negative control group were located at a considerable distance from the other pens in the hall.

The *C. perfringens* strain used in this experiment was obtained from the microbial bank of the Pasteur Institute of Iran (ATCC 13124). The lyophilized vial of *C. perfringens* (ATCC 13124) was first rehydrated and activated by inoculating into 10 mL of pre‐reduced cooked meat medium (prepared in‐house: HMH peptone B 98 g/L, proteose peptone 20 g/L, dextrose 2 g/L, sodium chloride 5 g/L, final pH adjusted to 7 at 25°C), followed by anaerobic incubation at 37°C for 24 h. The activated culture was then transferred to 1 L of fresh pre‐reduced cooked meat medium of the same composition and incubated under anaerobic conditions at 37°C for another 24 h to obtain the working bacterial suspension used in the experiment. All media were pre‐reduced prior to inoculation to maintain anaerobic conditions (Fathima et al. 2022a).

### 2.3 Probiotic Strains

In this study, a specific commercial multi‐strain probiotic, developed against *C. perfringens* and isolated in the laboratory of Nature Biotechnology Company (Biorun, Iran), was applied. It contained *Lactobacillus acidophilus, Pediococcus acidilactici, Lactobacillus rhamnosus, Bacillus subtilis, Bacillus megaterium* and *Bacillus amyloliquefaciens* with a minimum of 1 × 10^9^ CFU/g of the product.

### 2.4 Growth Performance

These parameters were measured on Days 1, 10 and 21 of the experiment following a 2‐h fasting period. Additionally, throughout the experiment, all mortalities were carefully recorded, including the number and weight of dead birds, along with detailed information for each experimental group. These data were essential for accurately calculating weight gain and feed intake, including adjustments for the chickens that died during the experiment.

### 2.5 Carcass Yield

On Day 21 of the experimental period, two male chickens were randomly selected from each replicate of every treatment group and slaughtered. The following parameters were measured: the relative weights of carcass yield, breast, thigh, liver, spleen, bursa of fabricius, proventriculus, gizzard and heart.

### 2.6 Evaluation of Gastrointestinal Tissue Damage (Ileum)

On Day 21, a 10‐cm segment of ileum tissue was isolated, and intestinal damage was evaluated using a scoring system ranging from 0 (completely healthy) to 4 (severe damage with infection) (Lee et al. 2013).

### 2.7 Enumeration of Gastrointestinal Bacteria and Measurement of Ileum Contents pH

Ileal samples were aseptically collected to determine bacterial populations, including enteric Gram‐negative bacteria, lactic acid bacteria (LAB), yeast, *C. perfringens*, *Bacillus* spp. and total aerobic bacteria.

For enumeration of enteric Gram‐negative bacteria, serial dilutions of ileal contents were plated on Eosin Methylene Blue (EMB) agar and incubated aerobically at 35°C ± 0.5°C for 18–24 h. LAB were enumerated using de Man, Rogosa and Sharpe (MRS) agar, followed by aerobic incubation at 35°C ± 0.5°C for 16–24 h. Yeasts were counted on Rose Bengal Chloramphenicol (RBC) agar after aerobic incubation at 25°C for approximately 72 h.

For *C. perfringens* enumeration, samples were cultured in pre‐reduced cooked meat medium and incubated under anaerobic conditions at 37°C for 24 h. Enumeration of *Bacillus* spp. was performed by plating on Tryptic Soy Agar (TSA) and incubating aerobically at 35°C ± 0.5°C for 16–24 h. Total aerobic bacteria were determined using nutrient agar, followed by aerobic incubation at 35°C ± 0.5°C for 16–24 h.

Additionally, to measure the pH of ileal contents, one gram of sample was mixed with 9 mL of distilled water in a Falcon tube and vortexed for 5 min, and the pH was measured using a pH meter.

### 2.8 Lipid Peroxidation

Lipid peroxidation, expressed as malondialdehyde (MDA) concentration of broiler thigh meat, was measured using the thiobarbituric acid‐reactive substances (TBARS) method as described by Ahn et al. (1999), with minor modifications. In brief, 5 g of thigh meat was homogenized with 30 mL of distilled water for 2 min. Then, 2 mL of homogenate was mixed with 4 mL of TCA/TBA (trichloroacetic acid/thiobarbituric acid) reagent (15% TCA [w/v] and 20 mM TBA) and 100 µL butylated hydroxyanisole (BHA). The mixture was incubated in a water bath at 95°C for 30 min, cooled to room temperature and centrifuged at 3000 rpm for 10 min. The absorbance of the supernatant was measured at 535 nm using a spectrophotometer. Lipid oxidation was expressed as TBARS value, reported as µg MDA per gram of fresh sample.

### 2.9 Intestinal Histomorphology

Sampling of duodenal, jejunal and ileal tissues for histological examination was performed by isolating the samples from the carcass, rinsing them with PBS solution, and immersing them in 10% formalin solution for 24 h. Subsequent processing included dehydration, clearing, paraffin embedding and sectioning into 5‐µm‐thick slices using a rotary microtome. After deparaffinization, tissue sections were stained with haematoxylin–eosin and Alcian blue. Finally, relevant morphological indices, such as villus height, villus width and crypt depth, were observed and measured under a light microscope at 40× magnification. Intestinal morphometric measurements were performed using an ocular graticule installed in one eyepiece of the microscope. For each slide, five well‐oriented villi with the greatest height were selected, and their villus height and villus width were measured accordingly (Kazemi et al. 2019).

### 2.10 Blood Biochemistry

On Day 20 of the experiment, two male chicks from each replicate of each treatment group were randomly selected. Blood samples were collected via wing vein puncture to analyse blood biochemical parameters, including cholesterol, HDL, LDL, triglycerides, glucose, total protein, albumin and globulin. The blood samples were centrifuged at 3000 × *g* for 15 min to separate plasma, which was then stored at −20°C until analysis. Plasma biochemical parameters were measured using a spectrophotometric method with commercial kits (Pars Azmun Co. Tehran, Iran) and a microplate reader (Stat Fax 3200; Awareness Technology Inc., Palm City, FL, USA), according to the manufacturers’ instructions.

### 2.11 Measurement of Short‐Chain Fatty Acids (SCFAs) in Cecum

For the analysis of SCFAs, including acetic acid, propionic acid, butyric acid and lactic acid in cecal contents, gas chromatography (GC; Agilent Technologies, USA) equipped with an HP‐FFAP capillary column was employed. Helium was used as the carrier gas. The injector and detector temperatures were set at 250°C and 300°C, respectively, and the total run time was 15 min. Quantification of SCFAs was performed using external standards of individual SCFAs. Cecal contents were collected from two birds per replicate on Day 20 and stored at −20°C in the laboratory until SCFAs analysis was conducted.

### 2.12 Statistical Analysis

The data from this experiment, structured as a completely randomized design with six treatments and five replicates, were analysed by one‐way ANOVA using the general linear model procedures (GLM) of SAS software (version 9.4). The significance level was set at 0.05, and mean comparisons were conducted using the Duncan's multiple range test.

$$Y_{ij}=\mu +t_{i}+e_{ij}$$

where *Y_ij_* represents the observation for the *j*th replicate of the *i*th treatment, *μ* represents the overall mean of the model, indicating the average response across all treatments and replications. *τ_i_* represents the fixed effect of the *i*th treatment, and *e_ij_* represents the error term associated with the *j*th replication of the *i*th treatment.

## 3 Results

### 3.1 Growth Performance

Table 3 shows the results for the effects of experimental groups on growth performance. Results indicated that administering probiotics and chelated zinc from the start of the rearing period improved broiler body weight during the first 10 days, prior to challenge initiation (*p* < 0.05). In this experiment, the challenge group generally showed poorer performance in terms of body weight compared to the control group throughout the overall rearing period (1–21 days) (*p* < 0.05). The challenge + probiotic and challenge + zinc groups seemed to mitigate some of the negative effects of the challenge, leading to better performance compared to the challenge group alone. The challenge + copper group did not seem to be as effective, showing similar results to the challenge group. The challenge + combination of probiotics, zinc and copper provided intermediate results, not as effective as the challenge + probiotic or challenge + zinc groups alone, but still better than the challenge group (*p* < 0.05). The results showed that dietary supplementation with chelated zinc significantly increased feed intake compared to the other treatment groups, particularly the control group (*p* < 0.05), during the grower period (11–21 days) and the entire rearing period (1–21 days). The findings suggested that zinc and probiotic supplementation in the presence of a challenge had a significant positive effect on growth performance compared to other groups, as indicated by both higher body weight gain and feed intake, coupled with a more efficient feed conversion ratio.

**TABLE 3:** The effects of experimental groups on growth performance of broiler chickens challenged with *Clostridium perfringens*

	Body weight (g)			Feed intake (g)			Feed conversion ratio		
Treatment	1–10 days	11–21 days	1–21 days	1–10 days	11–21 days	1–21 days	1–10 days g/g	11–21 days g/g	1–21 days g/g
Control	215.61^ab^	368.77	584.38^a^	282.46	640.31^b^	922.77^b^	1.310	1.738	1.580
Challenge	215.23^ab^	326.28	541.51^b^	281.07	675.99^ab^	957.07^ab^	1.307	2.097	1.776
Challenge + Probiotic	220.61^a^	360.74	581.35^a^	278.15	672.54^ab^	950.70^ab^	1.262	1.864	1.635
Challenge + Zinc	220.00^a^	364.55	584.55^a^	276.23	708.78^a^	985.02^a^	1.257	1.948	1.686
Challenge + Copper	205.00^b^	329.12	534.12^b^	276.76	633.03^b^	909.80^b^	1.351	1.932	1.705
Challenge + combination of probiotics, zinc, and copper	210.76^ab^	342.85	553.62^ab^	292.38	639.72^b^	932.11^b^	1.387	1.879	1.687
SEM	1.68	5.37	6.10	2.74	7.37	7.18	0.01	0.034	0.019
*p* Value	0.048	0.072	0.020	0.582	0.008	0.021	0.14	0.065	0.054

*Note*: In each column, least squares means with a different superscript letters (a,b) differ significantly (*p* < 0.05). Each mean is represented by 5 replications including 13 birds each.

Abbreviation: SEM, standard error of the mean (pooled standard error).

### 3.2 Carcass Yield

Table 4 presents the results regarding the impact of experimental groups on carcass traits. Significant differences were observed in breast weight among the treatment groups, suggesting that the challenge group had a detrimental effect on this parameter. However, supplementing the diet with chelated copper or a combination of probiotics, zinc and copper appeared to mitigate the negative impact of the challenge on breast weight. No significant differences were detected among the experimental groups for other parameters such as relative carcass weight, thigh, heart, spleen, liver, gizzard, proventriculus and bursa weights.

**TABLE 4:** The effects of experimental groups on relative weights of internal organs of broiler chickens challenged with *Clostridium perfringens*.

Treatment	Carcass (%)	Breast (%)	Thigh (%)	Heart (%)	Spleen (%)	Liver (%)	Gizzard (%)	Proventriculus (%)	Bursa (%)
Control	59.14	18.47^a^	15.01	0.63	0.08	2.72	2.44	0.59	0.22
Challenge	54.17	15.51^b^	15.62	0.61	0.12	2.74	2.92	0.75	0.22
Challenge + Probiotic	58.36	17.61^ab^	16.11	0.65	0.10	2.47	2.98	0.64	0.23
Challenge + Zinc	58.87	17.54^ab^	16.61	0.67	0.10	2.55	2.72	0.65	0.28
Challenge + Copper	58.92	18.53^a^	16.54	0.68	0.11	2.55	2.78	0.69	0.25
Challenge + Combination of probiotics, zinc, and copper	59.00	18.92^a^	17.06	0.59	0.09	2.45	2.57	0.63	0.25
SEM	0.59	0.34	0.25	0.01	0.004	0.04	0.06	0.01	0.01
*p* Value	0.09	0.040	0.21	0.06	0.11	0.34	0.16	0.19	0.53

*Note*: In each column, least square means with different superscript letters (a,b) differ significantly (*p* < 0.05). Each mean is represented by five replications two birds each.

Abbreviation: SEM, standard error of the mean (pooled standard error).

### 3.3 Intestinal Morphology

Table 5 shows the effects of experimental groups on intestinal morphology. The highest villus height in the duodenum was observed in the challenge + probiotic group, significantly different from all other groups. The challenge group had the lowest villus height (*p* = 0.002). The challenge + probiotic group also had the greatest villus width, significantly different from the other experimental groups. The challenge and challenge + copper groups showed the smallest villus widths (*p* = 0.02). Overall, the challenge + probiotic group appears most effective in mitigating the negative effects of the challenge, particularly in terms of villus height, width and crypt depth.

**TABLE 5:** The effects of experimental groups on intestinal morphology of 3‐week‐old broiler chickens challenged with *Clostridium perfringens*.

	Duodenum			
Treatment	Villus height (µm)	Villus width (µm)	Crypt depth (µm)	VH:CD
Control	1339.3^b^	148.6^b^	274.6	5.14
Challenge	1121.0^c^	114.3^c^	322.3	3.47
Challenge + Probiotic	1599.6^a^	166.0^a^	207.3	8.22
Challenge + Zinc	1408.6^b^	130.3^bc^	217.3	6.54
Challenge + Copper	1440.0^b^	112.6^c^	283.6	5.07
Challenge + Combination of probiotics, zinc and copper	1313.0^b^	130.6^bc^	23.43	6.22
SEM	38.4	5.7	14.4	4.9
*p* Value	0.002	0.02	0.13	0.07

	**Jejunum**			
Treatment	Villus height (µm)	Villus width (µm)	Crypt depth (µm)	VH:CD
Control	1040.0^a^	103.6	131.6^b^	7.93^a^
Challenge	711.3^b^	106.3	255.3^a^	2.89^d^
Challenge + Probiotic	987.7^a^	111.0	165.0^b^	6.43^abc^
Challenge + Zinc	1059.7^a^	116.3	211.0^ab^	4.79^c^
Challenge + Copper	969.3^ab^	115.0	164.6^b^	5.89^bc^
Challenge + Combination of probiotics, zinc and copper	1119.0^a^	11.36	14.46^b^	7.68^ab^
SEM	45.9	3.7	13.2	4.6
*p* Value	0.012	0.93	0.03	0.006

	**Ileum**			
Treatment	Villus height (µm)	Villus width (µm)	Crypt depth (µm)	VH:CD
Control	805.0^a^	144.6^ab^	181.6^a^	4.44
Challenge	611.6^b^	112.0^c^	257.0^b^	2.37
Challenge + Probiotic	787.6^a^	161.6^a^	182.0^a^	4.64
Challenge + Zinc	823.0^a^	125.6^bc^	192.6^a^	4.34
Challenge + Copper	791.0^a^	119.2^bc^	189.3^a^	4.18
Challenge + Combination of probiotics, zinc and copper	792.3^a^	135.3^abc^	173.6^a^	4.64
SEM	22.8	5.1	8.6	2.7
*p* Value	0.04	0.01	0.02	0.09

*Note*: In each column, least square means with a different superscript letters (a–c) differ significantly (*p* < 0.05). Each mean is represented by five replications two birds each.

Abbreviations: SEM, standard error of the mean (pooled standard error); VH:CD, villus height/crypt depth.

There were significant differences in crypt depth of the jejunum (*p* = 0.03). The challenge group showed the greatest crypt depth, indicating a negative impact. The challenge + probiotic, challenge + copper and challenge + combination of probiotics, zinc and copper significantly reduced crypt depth compared to the challenge group. There were significant differences in the VH ratio (*p* = 0.006). The control and challenge + combination of probiotics, zinc and copper had the highest ratios, whereas the challenge group showed the lowest, indicating a negative effect. Overall, the challenge + probiotic, challenge + zinc and challenge + combination of probiotics, zinc and copper appeared to mitigate the negative effects of the challenge on the jejunum, with the challenge + combination of probiotics, zinc and copper showing the most consistent positive impact across different parameters.

The villus height of the ileum was significantly lower in the challenge group compared to all other groups (*p* = 0.04). The challenge + probiotic, challenge + zinc, challenge + copper and challenge + combination of probiotics, zinc and copper showed villus heights similar to the control group, indicating that these treatments mitigated the detrimental effects observed in the challenge group. Villus width was significantly higher in the challenge + probiotic group compared to all other groups (*p* = 0.01). The challenge group had the smallest villus width. The challenge + zinc, challenge + copper and challenge + combination of probiotics, zinc and copper had intermediate values, not significantly different from the control group. Crypt depth was significantly higher in the challenge group compared to all other groups (*p* = 0.02). The challenge + probiotic, challenge + zinc, challenge + copper and challenge + combination of probiotics, zinc and copper resulted in crypt depths similar to the control group, indicating a restorative effect. The intestinal surface was significantly lower in the challenge group compared to all other groups (*p* = 0.01). The challenge + probiotic group had the highest intestinal surface, indicating a protective or restorative effect. The challenge + zinc and challenge + combination of probiotics, zinc and copper also showed higher values, whereas the challenge + copper group had intermediate values. Overall, the challenge + probiotic and challenge + combination of probiotics, zinc and copper appeared to be the most effective in mitigating the negative effects of the challenge on the ileum, with challenge + zinc also showing notable benefits.

### 3.4 Microbial Population of Ileum

Table 6 shows the effects of experimental groups on the microbial population of the ileum. These results indicate specific impacts of treatments on microbial populations, particularly in controlling potentially harmful bacteria like *C. perfringens* and promoting beneficial bacteria like *Bacillus* under certain conditions. There was a significant difference in *C. perfringens* count among the groups (*p* = 0.001). The challenge group had significantly higher *C. perfringens* counts compared to other groups. There was a significant difference in *Bacillus* counts among the groups (*p* = 0.004). The challenge + probiotic group had significantly higher *Bacillus* counts compared to other groups.

**TABLE 6:** The effects of experimental groups on microbial population of ileal microflora of broiler chickens challenged with *Clostridium perfringens*.

Treatment	Total (10^8^ CFU/g)	Gram‐negative (10^3^ CFU/g)	*Lactobacillus* (10^8^ CFU/g)	Yeast (10^3^ CFU/g)	*C. perfringens* (10^5^ CFU/g)	*Bacillus* (10^5^ CFU/g)
Control	38.62	1.93	2.01	3.71	1.27^d^	6.65^ab^
Challenge	18.00	2.45	1.03	3.37	14.00^a^	2.25^c^
Challenge + Probiotic	33.00	1.90	1.53	3.75	5.50^c^	7.65^a^
Challenge + Zinc	30.30	1.04	1.36	3.42	6.75^bc^	4.55^bc^
Challenge + Copper	25.12	2.67	1.52	4.05	7.90^b^	4.70^bc^
Challenge + Combination of probiotics, zinc and copper	29.00	1.53	1.54	3.78	6.67^bc^	6.57^ab^
SEM	2.46	0.27	0.11	0.23	0.82	0.48
*p* Value	0.23	0.60	0.32	0.97	0.001	0.004

*Note*: In each column, least square means with a different superscript letters (a–d) differ significantly (*p* < 0.05). Each mean is represented by five replications two birds each.

Abbreviation: SEM, standard error of the mean (pooled standard error).

### 3.5 Meat Oxidation, Ileum Contents Acidity, Ileum Tissue Damage Score and Mortality

Table 7 depicts the results for the effects of experimental groups on meat oxidation, ileum contents acidity, ileum tissue damage score and mortality. The control group consistently showed the most favourable outcomes in terms of oxidation, pH, tissue damage score and mortality. The challenge group exhibited the most adverse effects across all measured parameters. The challenge + probiotic, challenge + zinc, challenge + copper and challenge + combination of probiotics, zinc and copper generally mitigated some adverse effects compared to the challenge group, but not to the level of the control group. These comparisons highlight the varying impacts of different experimental groups on the measured parameters, indicating potential protective or exacerbating effects on the biological responses studied.

**TABLE 7:** The effects of experimental groups on lipid oxidation thigh meat, ileum contents acidity, ileum tissue damage score and mortality of 3‐week‐old broiler chickens challenged with *Clostridium perfringens*.

Treatment	Malondialdehyde oxidation (µg/g)	Ileal pH	Ileal lesions score^1^ (score 0–4)	Mortality (% of treatment)
Control	1.78^d^	5.52^d^	0.20^d^	0.00^b^
Challenge	3.10^a^	6.34^a^	2.80^a^	6.15^a^
Challenge + Probiotic	2.71^c^	5.78^c^	1.60^bc^	1.53^b^
Challenge + Zinc	2.65^c^	6.10^ab^	1.60^bc^	3.07^ab^
Challenge + Copper	2.94^b^	6.26^a^	2.40^b^	1.53^b^
Challenge + Combination of probiotics, zinc and copper	2.91^b^	5.98^bc^	1.40^c^	0.00^b^
SEM	0.08	0.07	0.18	0.01
*p* Value	0.001	0.001	0.001	0.025

*Note*: In each column, least square means with a different superscript letters (^a–d)^ differ significantly (*p* < 0.05). Every mean is represented by five replications two birds each.

Abbreviation: SEM, standard error of the mean (Pooled standard error).

^1^Ileal lesions score were reported on Day 21 on a 0–4 score and analysed with Lee et al. (2013) method.

### 3.6 Blood Biochemical Parameters

Table 8 illustrates the results of the effects of experimental groups on blood biochemical parameters. The highest albumin level was observed in the challenge + probiotic group, significantly higher than all other groups. The differences were statistically significant (*p* = 0.004), indicating a notable increase in albumin with the challenge + probiotic group. The control and challenge groups have significantly higher glucose levels compared to challenge + combination of probiotics, zinc and copper. The differences were statistically significant (*p* = 0.006), indicating lower glucose levels with challenge + combination of probiotics, zinc and copper. The challenge + zinc and challenge + combination of probiotics, zinc and coppers had slightly lower levels. The differences were statistically significant (*p* = 0.01), indicating variations in total protein levels among groups. The challenge + combination of probiotics, zinc and copper had significantly lower HDL levels compared to control and other experimental groups. The differences were statistically significant (*p* = 0.01), indicating variations in total protein levels among groups. The challenge + combination of probiotics, zinc and copper had significantly lower HDL levels compared to control and other groups. The differences were statistically significant (*p* = 0.001), indicating lower HDL levels with challenge + combination of probiotics, zinc and copper.

**TABLE 8:** The effects of experimental groups on blood biochemical parameters of 3‐week‐old broiler chickens challenged with *Clostridium perfringens*.

Treatment	Albumin (g/dL)	Cholesterol (mg/dL)	Glucose (mg/dL)	Triglycerides (mg/dL)	Total protein (g/dL)	Uric acid (mg/dL)	HDL (mg/dL)	LDL (mg/dL)	Globulin (g/dL)
Control	2.64^bc^	137.72	287.14^a^	86.75	3.64ab	6.15	66.96^a^	123.04^a^	0.99
Challenge	2.70^b^	137.99	284.71^a^	84.12	3.98^ab^	6.68	67.20^a^	88.00^bc^	1.27
Challenge + Probiotic	3.28^a^	129.49	274.51^ab^	65.45	4.31^a^	5.59	58.56^ab^	69.08^c^	0.98
Challenge + Zinc	2.12^cd^	137.38	244.85^bc^	80.93	3.28^b^	7.38	57.60^ab^	80.08^bc^	1.15
Challenge + Copper	1.97^d^	130.52	233.11^c^	83.55	3.31^b^	6.35	52.32^bc^	91.84^bc^	1.33
Challenge + Combination of probiotics, zinc and copper	2.24^bcd^	125.59	228.30^c^	70.99	3.27^b^	7.81	47.04^c^	102.56^ab^	1.02
SEM	0.10	2.43	6.54	2.71	0.11	0.29	1.82	4.44	0.07
*p* Value	0.004	0.59	0.006	0.13	0.01	0.25	0.001	0.002	0.71

*Note*: In each column, least square means with a different superscript letters (^a–d^) differ significantly (*p* < 0.05). Every mean is represented by five replications two birds each.

Abbreviation: SEM, standard error of the mean (pooled standard error).

### 3.7 Short‐Chain Fatty Acids

The results of the effect of experimental groups on the cecal short chain fatty acid composition of broilers are shown in Table 9. The results showed that the addition of probiotics increased the short chain fatty acid composition, whereas *C. perfringens* reduced the short chain fatty acid composition compared to the control group. For example, probiotic supplementation increased acetate, propionate and butyrate levels compared to other groups under *C. perfringens* challenge.

**TABLE 9:** The effects of experimental groups on short chain fatty acid composition of 3‐week‐old broiler chickens challenged with *Clostridium perfringens*.

Treatment	Acetate (µg/g)	Propionate (µg/g)	Isobutyrate (µg/g)	Butyrate (µg/g)	Isovalerate (µg/g)	Valerate (µg/g)
Control	38.88^a^	11.02^a^	0.63^a^	7.67^a^	0.069^ab^	0.066
Challenge	33.45^a^	8.01^b^	0.46^bc^	3.06^b^	0.074^a^	0.051
Challenge + Probiotic	40.61^a^	10.86^a^	0.57^ab^	5.98^a^	0.076^a^	0.063
Challenge + Zinc	25.56^b^	5.53^c^	0.39^c^	1.93^b^	0.04^b^	0.024
Challenge + Copper	22.50^b^	5.12^c^	0.45^b^ ^c^	3.09^b^	0.039^b^	0.050
Challenge + Combination of probiotics, zinc and copper	37.38^a^	9.98^ab^	0.61^a^	6.28^a^	0.064^ab^	0.065
SEM	1.79	0.64	0.026	0.54	0.004	0.005
*p* Value	0.004	0.003	0.01	0.002	0.039	0.19

*Note*: In each column, least square means with different superscript letters (a–c) differ significantly (*p* < 0.05). Every mean is represented by five replications two birds each.

Abbreviation: SEM, standard error of the mean (pooled standard error).

## 4 Discussion

This study was conducted to investigate the effects of multi‐strain probiotics, zinc and copper on the performance of broiler chickens challenged with *C. perfringens*. The results indicated that *C. perfringens* significantly reduced body weight compared to the control group. These findings are consistent with previous reports (Li et al. 2018; Zaytsoff et al. 2020; Mahdi Raeisol Sadati et al. 2024). The presence of pathogenic *C. perfringens* species in the intestines can trigger an immune response and inflammation (Sun and Hirota 2015), diverting energy and nutrients away from growth toward the immune system, thus resulting in slower weight gain (Kanai et al. 2015). Moreover, *C. perfringens* species can produce toxins that damage the intestinal mucosa (Guo et al. 2020), impairing the digestive tract's ability to efficiently absorb nutrients from feed and leading to poor feed efficiency and, consequently, a reduction in body weight.

The results indicated that probiotic supplementation significantly enhanced body weight, aligning with findings from other studies on the effects of probiotics under challenging conditions (Ritzi et al. 2014; Gobi et al. 2018). This improvement in body weight could be attributed to the beneficial effects of probiotics on intestinal morphology, as well as on the populations of *C. perfringens* and *Lactobacillus*, as demonstrated in the present study. This finding suggests that the inclusion of probiotics in the chickens’ diet stimulates appetite and enhances digestion and nutrient absorption, resulting in increased feed consumption compared to those not receiving probiotics. During challenging conditions, such as exposure to pathogens or environmental stressors, animals may experience alterations in digestive efficiency and nutrient uptake, which probiotics can help mitigate.

Our findings suggest that dietary inclusion of chelated zinc positively influences growth performance in broilers, as evidenced by notable improvements in body weight and feed intake both prior to and following the challenge phase. This improvement could be attributed to the enhanced bioavailability of chelated zinc sources compared to inorganic ones, which may contribute to more efficient nutrient utilization. Consistent patterns have been reported in earlier studies, where supplementation with chelated zinc was associated with increased feed consumption and body weight gain relative to unsupplemented birds (Zhao et al. 2016; Wang et al. 2016; Ao et al. 2006).

One possible explanation for this response may be the favourable impact of chelated zinc on digestive efficiency and nutrient absorption within the gut. Given zinc's essential involvement in metabolic pathways, particularly those related to protein and carbohydrate metabolism, it is plausible to suggest that chelated zinc enhances gastrointestinal function, ultimately supporting better growth outcomes (Zhu et al. 2022; Bao et al. 2009). This interpretation is consistent with the findings of Chang et al. (2023), who also reported that zinc supplementation resulted in increased feed intake and weight gain.

Carcass yield, defined as the eviscerated carcass weight expressed as a percentage of pre‐slaughter live weight, represents a critical parameter for evaluating meat production efficiency in broiler chickens. According to Jahanian et al. (2008) and Khan et al. (2024), dietary supplementation with chelated zinc at 120 and 80 mg/kg, respectively, significantly enhanced breast meat yield and improved growth performance. Moreover, previous studies have indicated that under pathological conditions such as *C. perfringens* infection, intestinal inflammation suppresses nutrient absorption and impairs the development of both muscle and visceral organs (Mora et al. 2020).

Additionally, immune activation redirects amino acids toward the synthesis of acute‐phase proteins rather than supporting muscle accretion in the breast or thighs (Kumar et al. 2021).

Taken together, these mechanisms contribute to a reduction in relative carcass yield, particularly in relative breast weight, in poultry challenged with *C. perfringens*. Indeed, Mora et al. (2020) reported that carcass weight alterations resulting from *C. perfringens* challenge were predominantly confined to the breast and thigh tissues.

The results of this study also indicated that the additives used had no significant effect on other carcass yield parameters. This observation is consistent with previous reports suggesting that most additives primarily act luminally through mechanisms such as acidification, bacteriolysis and competitive exclusion and do not significantly influence systemic protein turnover or lipid deposition, which are key determinants of carcass yield (Younis et al. 2024).

The reduction in body weight can be attributed to changes in intestinal morphology. Villus height and width (critical factors in the structure of the small intestine that directly influence nutrient absorption) were adversely affected by the *C. perfringens* challenge (Metzler‐Zebeli et al. 2018; Daneshmand et al. 2022). As previously mentioned, *C. perfringens* is known to produce enterotoxins or cytotoxins, which directly damage the epithelial cells lining the villi in the small intestine (Navarro et al. 2018). Additionally, *C. perfringens* infection induces inflammation in the intestine, resulting in tissue damage, including the villi, which compromises their structure and function (Fu et al. 2022).

The findings of this study suggest that probiotic supplementation mitigates the intestinal damage caused by *C. perfringens* infection, primarily through improvements in villus structure and epithelial integrity. These morphological changes, including increased villus height and reduced crypt depth, indicate enhanced absorptive capacity and epithelial renewal (Naeem and Bourassa 2025). Beyond morphological improvements, probiotics exert multifaceted functional roles that collectively suppress *C. perfringens* proliferation and restore intestinal homeostasis (Zhao et al. 2022). Such improvements in intestinal architecture may contribute to better nutrient utilization and partially explain the improved performance observed in probiotic‐treated birds (Kulkarni et al. 2022; La Ragione et al. 2004; Zhao et al. 2022).

One of the primary mechanisms by which probiotics counteract *C. perfringens* is competitive exclusion, whereby beneficial microorganisms compete with pathogens for adhesion sites and nutrients within the intestinal lumen (Jiang et al. 2022). *Bacillus* species, particularly *B. subtilis* and *Bacillus licheniformis*, demonstrate strong anti *C. perfringens* activity through the production of antimicrobial compounds such as *lichenicidin* (La Ragione et al. 2004). This compound forms pores in bacterial membranes and inhibits cell wall biosynthesis. Consequently, this process results in increased villus length and decreased crypt depth in the small intestine, thereby preservation of intestinal villus structure and epithelial integrity (Zhao et al. 2022).

Studies demonstrate that probiotics, such as *Lactobacillus*, significantly improve gut morphology by increasing villus height in the ileum (Yosi and Metzler‐Zebeli 2023; Naeem and Bourassa 2025). In addition to their structural benefits, LAB modulate the intestinal environment through the production of organic acids and bacteriocins, which collectively inhibit the growth of *C. perfringens* (Zhou et al. 2021). The increased villus height provides a larger surface area for nutrient absorption, directly contributing to improved digestive efficiency and overall gut health (Awad et al. 2009).

Li et al. (2015) demonstrated that zinc mitigates intestinal inflammation via epigenetic pathways by regulating the expression of cytokines involved in inflammatory responses. They reported that chelated zinc upregulated IL‐10 and downregulated IL‐8 expression in broilers infected with *C. perfringens* and *Eimeria* spp., suggesting that chelated zinc helped attenuate jejunal inflammation in infected birds compared to control group. This reduction in inflammatory signalling may explain the increased villus height and decreased intestinal permeability observed in the chelated zinc‐supplemented group, which was associated with reduced mortality and improved performance.

The results also revealed a reduced population of *Bacillus* species in the challenged groups. *Bacillus* spp. play a vital role in maintaining gut microbial balance by outcompeting pathogenic bacteria for nutrients and colonization sites (Galleher et al. 2020). A reduction in beneficial bacterial populations disrupts microbial equilibrium, leading to dysbiosis and facilitating the overgrowth of opportunistic pathogens such as *C. perfringens* (Gong et al. 2021). This microbial imbalance compromises gut health, impairs nutrient absorption and negatively impacts growth performance and body weight (Galleher et al. 2020). Furthermore, the population of *C. perfringens* was significantly elevated in challenged broiler chickens, supporting the occurrence of dysbiosis and disease progression.

Consistent with our findings, Feng et al. (2022) reported that the typical population of *C. perfringens* in the ileum is generally below 10^5^ CFU/g of digesta. However, during necrotic enteritis, this number increases markedly. In subclinical cases, *C. perfringens* counts may reach approximately 10^7^ CFU/g, which is associated with impaired growth, mild intestinal lesions and low mortality. In clinical cases, the bacterial load may escalate to 10^9^ CFU/g, leading to extensive intestinal damage and significantly higher mortality rates. Probiotic supplementation has been shown to limit this pathological expansion by stabilizing the intestinal microbiota and preventing excessive pathogen colonization.

Consistent with the results of the present study, it was reported that zinc supplementation has been shown to lower pro‐inflammatory cytokine expression and positively influence the ileal microbiota, which can help suppress pathogenic bacteria (Bortoluzzi et al. 2020). Previous research highlights that supplementation with chelated zinc effectively mitigates intestinal inflammation by suppressing the expression of key pro‐inflammatory cytokines such as IL‐8, IFN‐γ, TLR‐2 and iNOS. Additionally, chelated zinc has been found to strengthen the intestinal barrier by reducing the permeability disruptions commonly induced by *C. perfringens* infection, particularly when combined with coccidiosis (Ng et al. 2025). These combined effects likely contribute to lowering the colonization levels of *C. perfringens* within the small intestine, thereby supporting improved gut health under pathogenic challenges (Bortoluzzi et al. 2019).

In this regard, it has been reported that copper, when used at pharmacological levels in broiler diets, exhibits antibacterial properties. This leads to improved growth performance, a reduction in pathogenic bacteria and enhanced nutrient absorption (Santos et al. 2020). Moreover, research indicates that increasing dietary copper levels up to 250 mg/kg leads to a progressive reduction in *E. coli* populations within the ileal digesta of broilers. This observation highlights a direct relationship between copper supplementation and modulation of intestinal bacterial communities, underscoring the important role of copper in maintaining gut microbial balance (Pang et al. 2009).

According to the results of this study, the challenge group exhibited higher lesion scores, confirming the negative effects of *C. perfringens* on intestinal health. These results demonstrate the detrimental impact of *C. perfringens* on performance and overall gut function in broilers.

However, studies have demonstrated that probiotic supplements can counteract the detrimental effects of intestinal pathogens. Probiotics improve villus height and width by strengthening the intestinal barrier through mechanisms such as promoting mucin production (Mohammed et al. 2024), tightening epithelial cell junctions, reducing inflammation (Al‐Baadani et al. 2016) and preventing damage to intestinal tissues (Zhao et al. 2020). Furthermore, probiotics produce SCFAs like butyrate, which are essential for intestinal health. Butyrate, in particular, serves as a primary energy source for enterocytes and plays a critical role in reinforcing tight junction integrity, thereby strengthening the intestinal barrier against pathogen invasion. Consequently, probiotics indirectly protect intestinal tissues by enhancing epithelial resilience and reducing pathogen‐induced damage (Calik and Ergün 2015). Therefore, probiotics play a crucial role in enhancing intestinal morphology under challenging conditions by mitigating the negative impacts of pathogens like *C. perfringens* and supporting overall gut health.

Zinc supplementation has been shown to enhance intestinal integrity and modulate inflammatory responses by downregulating pro‐inflammatory cytokine expression in broilers challenged with *C. perfringens* (Bortoluzzi et al. 2020; Mathis et al. 2020). These combined effects help alleviate intestinal inflammation and ultimately contribute to reduced tissue damage during enteric infections.

Consistent with our findings that copper supplementation reduces intestinal tissue damage caused by *C. perfringens*, previous studies have reported that copper improves intestinal lesion scores during infection by enhancing nutrient absorption in the duodenum, strengthening immune responses, maintaining gut health and increasing resistance against enteric pathogens (Santos et al. 2020; Chen et al. 2023).

As observed in the present study and supported by previous reports, *C. perfringens* primarily elevates intestinal pH in poultry through amino acid fermentation, a metabolic process associated with ammonia production (Stanley et al. 2012). This ammonia, as a product of protein catabolism and inherently alkaline in nature, directly contributes to pH elevation by neutralizing acidic components within the gut lumen (Jakhar et al. 2024). Furthermore, challenge with *C. perfringens* alters gut microbiota composition by suppressing LAB and limiting the growth of beneficial genera such as *Bacillus* and *Lactobacillus*. As LAB contribute to intestinal acidification via lactic acid production, their reduction may decrease acid levels and consequently elevate intestinal pH (Józefiak et al. 2014; Liu et al. 2010).

Consistent with the present study, the use of probiotics in poultry has been widely reported to lower intestinal pH, even in the presence of *C. perfringens* infection. This acidification effect is primarily mediated by the microbial production of SCFAs, including acetate, propionate and butyrate, which create an unfavourable environment for *C. perfringens* proliferation. Lower intestinal pH directly inhibits toxin production and vegetative growth of *C. perfringens* (Zhang et al. 2020).

The results also confirmed elevated levels of meat oxidation in the challenge group compared to the other groups, aligning with findings from previous studies (Liao et al. 2015; Zhang et al. 2024). Chronic inflammation induced by *C. perfringens* has been reported to increase pro‐inflammatory markers such as TNF‐α and IL‐6. This inflammatory response in poultry may contribute to oxidative stress and muscle damage, ultimately leading to reduced meat quality (Al‐Nasser et al. 2024). Probiotic supplementation may help reduce lipid oxidation in meat by modulating gut microbiota and enhancing the animal's antioxidant status (Deng et al. 2022). Similarly, zinc supplementation has been associated with improved antioxidant capacity in animals, which, in turn, can reduce meat oxidation (Saleh et al. 2018). Moreover, adequate copper intake is essential for maintaining antioxidant enzyme activity, thereby helping to mitigate oxidative processes in meat (Wu et al. 2020).

The results demonstrated that probiotic supplementation could increase serum albumin and total protein concentrations, aligning with findings from other studies (Yazhini et al. 2018; Adriani et al. 2021). Probiotics achieve this effect by improving gut health, modulating immune function, producing beneficial metabolites and regulating protein synthesis, thereby contributing to better overall health. Probiotic supplementation also decreased serum LDL concentrations (Panda et al. 2006; Ashayerizadeh et al. 2011), potentially by enhancing LDL receptor expression in the liver, which increases LDL cholesterol clearance from the blood. Probiotics improve liver function by reducing harmful metabolites such as ammonia and MDA, while enhancing the activity of antioxidant enzymes, including glutathione peroxidase and superoxide dismutase. These effects help create a more favourable environment for albumin synthesis (Derakhshan et al. 2023; Lebedev et al. 2024).

Additionally, zinc and copper supplementation were shown to decrease serum glucose concentrations, consistent with findings from previous studies on zinc (Yalçinkaya et al. 2012; Faghih‐Mohammadi et al. 2023) and copper (El‐Hady and Mohamed 2019). Studies have shown that copper proteinate supplementation can reduce plasma triglycerides and cholesterol levels, including HDL, compared to inorganic copper sources (Ferreira Júnior et al. 2022). This reduction in HDL cholesterol may be associated with improved lipid metabolism and enhanced antioxidant status in broiler chickens fed chelated copper sources (Jegede et al. 2011).

The liver senses plasma Zn/Cu levels and adjusts gene expression accordingly. When metals arrive in stable organic complexes, less albumin is needed for transport, leading to reduced albumin mRNA and increased metallothionein for storage (Wen et al. 2019). This results in slightly lower serum albumin without affecting protein nutrition. Consistently, studies have shown that dietary chelated zinc or copper supplementation can reduce serum albumin or total protein levels, without negative effects on growth, feed efficiency or overall health, supporting this regulatory mechanism (Sarvari et al. 2015; Wang et al. 2019).

A study has indicated that the use of probiotic can enhance intestinal function and promote a balanced gut microbiota, both of which play a critical role in the production of SCFAs (Min et al. 2024). In broiler chickens, dietary supplementation with probiotic has been shown to improve intestinal health and create a favourable environment for the growth of beneficial microorganisms involved in SCFA synthesis (Wang et al. 2024). Additionally, probiotic compounds can strengthen both cellular and humoral immune responses and enhance antioxidant capacity, thereby reducing intestinal inflammation and supporting the maintenance of a stable and efficient microbiota for SCFA production (Wang et al. 2021).

*Lactobacillus* species, which are among the main producers of SCFAs in the broiler gut, may exhibit sensitivity to high levels of zinc and copper (Leonardi et al. 2013). Exposure to copper has been reported to result in the complete elimination of beneficial strains such as *Lactobacillus johnsonii*, leading to reduced acetate and butyrate production, which is associated with a marked decrease in total SCFA levels (Wen et al. 2023). In addition, copper and zinc supplementation has been shown to reduce the abundance of key fibre‐fermenting bacterial populations (Du et al. 2023). It has been reported that copper supplementation has been reported to reduce SCFA concentrations, particularly butyrate, which correlates with a decreased abundance of fatty acid‐producing bacteria (Du et al. 2023).

## 5 Conclusions

In conclusion, this study highlights the detrimental impact of *C. perfringens* infection on growth performance and intestinal health of broiler chickens, evidenced by reduced body weight, poor feed efficiency and compromised gut morphology. The infection resulted in an increased population of *C. perfringens* bacteria and a reduction in beneficial *Lactobacillus* species, resulting in dysbiosis and impaired nutrient absorption. However, the supplementation of multi‐strain probiotics, zinc and copper conferred significant benefits, improving body weight, feed conversion ratio and overall gut health. Probiotics, in particular, were effective in counteracting the adverse effects of *C. perfringens* by enhancing villus structure, promoting a balanced gut microbiota and improving nutrient absorption and feed intake. A mixture of copper, zinc and probiotics exhibited synergistic effects, including increased SCFA concentrations, ileal villus height and width and breast muscle yield, while reducing glucose levels, ileal lesion scores and mortality. Additionally, zinc and copper contributed to improved antioxidant capacity and supported metabolic functions. These findings underscore the importance of dietary interventions, particularly probiotics, in managing gut health and performance in broiler chickens under pathogenic challenges. Future research should aim to elucidate the precise mechanisms underlying these effects, potentially leading to optimized nutritional strategies for enhancing poultry health and productivity.

## Author Contributions

**Amin Kazemi**: investigation, formal analysis, writing – original draft. **Hassan Kermanshahi**: conceptualization, supervision, validation, writing – review and editing. **Reza Majidzahe Heravi**: conceptualization, methodology, validation, writing – review and editing. **Gholamreza Salehi Jouzani**: visualization, validation, writing – review and editing. **Ali Javadmanesh**: formal analysis, validation, writing – review and editing. All authors have read and approved the final manuscript.

## Funding

The authors would like to thank Ferdowsi University of Mashhad for their Financial support of the study.

## Ethics Statement

At Ferdowsi university of Mashhad, we believe that ensuring the well‐being of our poultry is paramount to ethical farming and quality production. We are committed to implementing the highest standards of animal welfare throughout our operations. The authors confirm that the ethical policies of the journal, as noted on the journal's author guidelines page, have been adhered to and the appropriate ethical review committee approval has been received. In this study, all the ethical standards for working on broilers obtained according to the approval of the Ethics Committee of Ferdowsi University of Mashhad. We conducted this experiment under the supervision of the Ethics Committee of Ferdowsi University of Mashhad with code 136.

## Conflicts of Interest

The authors declare no conflicts of interest.

## Data Availability

The data that support the findings of this study are available on request from the corresponding author. The data are not publicly available due to privacy or ethical restrictions.
